# Long-Term Efficacy of Bilateral Globus Pallidus Internus Deep Brain Stimulation in Myoclonus-Dystonia Associated with KCNN2 Gene Mutation: A Case Study

**DOI:** 10.3390/ijms26167736

**Published:** 2025-08-10

**Authors:** Olga Stodulska, Lukasz Milanowski, Dariusz Koziorowski, Tomasz Mandat, Stanislaw Szlufik

**Affiliations:** 1 Department of Neurology, Faculty of Health Sciences, Medical University of Warsaw, Ul. Zwirki i Wigury 61, 02-091 Warsaw, Poland; 2 Department of Neurosurgery, Maria Sklodowska-Curie National Research Institute of Oncology, 02-781 Warsaw, Poland

**Keywords:** dystonia-myoclonus, deep brain stimulation, GPi-DBS, KCNN2 mutation, myoclonus-dystonia syndrome

## Abstract

Dystonia-myoclonus syndrome is a rare neurological condition characterized by involuntary muscle contractions and myoclonic jerks, significantly impairing daily functioning. Pharmacological management is often ineffective, prompting consideration of alternative therapeutic interventions such as deep brain stimulation (DBS). This report describes a novel clinical case involving a 38-year-old female with severe dystonic and myoclonic symptoms associated with a pathogenic mutation in the KCNN2 gene (DYT34). Bilateral DBS targeting the internal segment of the globus pallidus (GPi) resulted in marked and sustained symptom improvement, notably reducing dystonic posturing and myoclonic movements over the 24-month follow-up period. Neuropsychological and neurologopedic assessments revealed no adverse effects on cognition or speech. This represents the first sufficient effect of GPi-DBS in a patient with a genetically confirmed KCNN2 mutation, highlighting its potential efficacy and underscoring the need for genetic testing in patients presenting with dystonia-myoclonus syndromes.

## 1. Introduction

Dystonia is a neurological disorder characterized by increased muscle tension leading to involuntary, paroxysmal, and twisting movements. Prolonged muscle tension ultimately results in the adoption of unnatural body postures. Dystonia encompasses a heterogeneous group of conditions, including primary dystonia (caused by genetic mutations) and secondary forms (iatrogenic, toxic, or post-traumatic dystonia). Myoclonic dystonia is a subtype often determined by genetic factors, presenting with both dystonic and myoclonic movements (sudden, jerky, irregular, and short-lasting) [1].

There is a lack of definitive scientific evidence supporting the efficacy of pharmacological treatment in primary dystonia. Treatment typically commences with the oral administration of levodopa, as this can identify rare types of levodopa-responsive dystonia (DYT5 and DYT14). Efforts have also been made to treat dystonia using anticholinergic drugs, baclofen, and benzodiazepines. In the management of focal dystonia, denervation of the overactive muscle can be achieved by severing the nerve, the muscle itself, or by administering intramuscular injections of botulinum toxin [2]. Currently, the latter method is preferred because of its minimal invasiveness, repeatability, and localized effect. Deep brain stimulation (DBS) is considered in patients who do not achieve satisfactory improvement with pharmacological treatment and/or who have not experienced satisfactory improvement with increasing doses of botulinum toxin or altering injection schemes and who do not exhibit cognitive impairment or depressive symptoms [3,4]. DBS involves surgical placement of stimulating electrodes in the deep structures of the brain. In dystonia, hyperactivity of the motor cortex, premotor and supplementary motor areas, and cerebellum are observed at rest. DBS restores the balanced activity of these centers to levels comparable to those in healthy individuals [5]. For dystonia, the greatest efficacy is achieved by placing electrodes in the ventromedial portion of the internal segment of the globus pallidus (Globus Pallidus Internus, GPi) [6]. Another advantage of DBS is its ability to affect multiple muscle groups simultaneously, as observed in generalized or multifocal dystonia, where it is impractical to inject all affected areas with botulinum toxin [7]. A noticeable improvement in dystonia patients who underwent the DBS Gpi procedure should appear within a few hours after the stimulator is activated, but full effects may develop over several days or weeks [8]. According to some authors, the effects of DBS in dystonia may persist for up to 10 years [9].

## 2. Case Report

A 38-year-old female patient was admitted to the Department of Neurology for evaluation of potential surgical intervention to address involuntary movements in the upper limbs. These movements were initially observed at the age of 9. During her high school years, she began experiencing involuntary movements in the neck, accompanied by a twisting sensation, which progressively worsened and were intermittently accompanied by sudden, jerky movements. The symptoms improved with alcohol consumption but exacerbated following coffee intake. The initial diagnosis was cervical dystonia, and she commenced treatment with botulinum toxin injections administered every 3–4 months into selected neck muscles, based on the distribution pattern. However, despite multiple treatment attempts, including those guided by ultrasound, satisfactory improvement was not achieved. The patient has a positive family history, as her mother and her mother’s twin sister experienced “no-no” type tremors in the hands and head. During her stay in the department, neurological examination revealed dystonic movements of the head and upper limbs with concomitant myoclonus (mainly involving the head, neck, and upper limbs, and to a lesser extent, the lower limbs). The symptoms had been increasing in severity over the past few years and were now significantly impairing daily activities, including professional work. A brain MRI revealed a few demyelinating changes, although oligoclonal bands in CSF and antibodies against Borrelia burgdorferi (both in CSF and blood serum) were not present. Neurologopedic examination did not reveal any abnormalities in speech or swallowing. Handwriting was clearly impaired due to myoclonic tremor of the upper limbs. Neuropsychological testing found no cognitive impairment or anxiety-depressive symptoms. Physiotherapeutic assessment excluded static and dynamic balance disorders. Based on these findings, no contraindications for the procedure were found, and the patient was therefore qualified for bilateral DBS-GPi treatment. The patient underwent bilateral GPi-DBS surgery. The procedure was performed using microrecording and macrostimulation (Leadpoint^®^, Medtronic, Minneapolis, MN, USA) and permanent electrodes (3389-28, Medtronic), which were connected to internal pulse generators (Activa SC, Medtronic). The procedure was conducted without complications. Another hospitalization at the Neurology Department took place 1 month after the bilateral implantation of DBS-GPi electrodes, for the purpose of initiating stimulation. In the neurological examination, symptoms of similar severity to those during the previous visit were observed. After activating the stimulator, a significant reduction in cervical dystonia as well as myoclonus in the upper limbs was achieved; however, some dystonic tremor in the upper limbs persisted. Follow-up neuropsychological and neurologopedic tests did not show any worsening of cognitive abilities, presence of mood disorders, or deterioration of speech. Writing in both phases remained partially illegible. The patient was monitored at 6, 12, and 24 months after the procedure to assess the stimulation. A significant reduction in the symptoms of cervical and upper limb dystonia, as well as resolution of cervical and upper limb myoclonus, was observed. In the neuropsychological examination after 24 months, no significant worsening of cognitive impairment was noted. The neurologopedic examination also showed no marked deterioration in speech.

Given the presence of involuntary movements in both the patient’s mother and her mother’s twin sister, the early manifestation of symptoms, and the exclusion of toxic or traumatic factors, the patient underwent a genetic testing panel according to Department of Medical Genetics, Institute of Mother and Child pipeline, VEP2.7. This panel encompassed the 47 most common genes associated with mutations leading to dystonia and Parkinson’s disease (Supplementary Table S1) and was performed on the SureSelect Human All Exon v6 enrichment, Illumina NovaSeq 6000 platform. However, no abnormal variants were identified in any of the analyzed genes. The next step was whole-exome sequencing (WES), which revealed the heterozygous KCNN2 c.583C>T mutation (KCNN2 p.[(Gln195Ter)];[=]). This mutation is not annotated in gnomAD. Variants in this gene have been reported in individuals diagnosed with myoclonic dystonia with tremor (DYT34) [10]. The cosegragation of the variant in patient’s family members, who have also experienced involuntary movements, has not yet been established.

## 3. Discussion

The KCNN2 gene encodes a protein that constitutes a small conductance calcium-activated potassium channel (SK2), which is an essential component of the neuronal membrane and plays a critical role in regulating neuronal excitability. Consequently, gene expression is observed in the brain, particularly within the cerebellum. The gene is located on the long arm of chromosome 5 (5q22.3). Mutations in KCNN2 are implicated in two dominantly inherited disorders: myoclonic dystonia type 34 (DYT34) [10] and neurodevelopmental disorders with or without movement disorders or behavioral disturbances [11,12]. The initial report of patients with myoclonic dystonia and tremor associated with a KCNN2 gene mutation was documented relatively recently, in 2020, in the United Kingdom [10]. A family was identified with dystonia affecting the upper limbs, accompanied by myoclonus, tremor, oculomotor disturbances, and balance disorders. In this family, a KCNN2 gene mutation—substitution c.1112G>A resulting in p.Gly371Glu—was detected. Each of the five reported patients exhibited a distinct combination of the aforementioned symptoms with individually varying severity. A KCNN2 mutation, albeit at a different locus, was also identified in a family diagnosed with essential tremor [12]. Additionally, a case of a de novo mutation in a location proximal to that of our patient (c.581dupA resulting in p.Leu195Valfs*10) was reported, presenting with developmental delay, epilepsy, spasticity, cerebellar ataxia, and dystonia [13]. However, the authors posited that a single defective gene copy does not alter the protein’s function, and in murine experiments with this mutation, no behavioral changes were observed. Furthermore, in this patient, a homozygous in-frame deletion in ZNF135 was identified, which may have exerted a more significant influence on the clinical presentation. Among other types of myoclonic dystonia with a similar clinical profile, it is crucial to differentiate dystonia type 11 (DYT 11), caused by a mutation in the SGCE gene at locus 7q21.3 [14], and dystonia type 15 (DYT 15), with a mutation at locus 18p11 [15]. To date, there have been no documented successful attempts in the literature to treat dystonia type 15 or type 34 using DBS. The only previous paper reports insufficient effect of DBS treatment in KCNN2 gene patient [16]. However, DBS treatment in patients with SGCE dystonia is demonstrated to lead to a significant reduction in both dystonic symptoms and myoclonus. In most of these cases, a bilateral implantation of electrodes into the internal segment of the globus pallidus (GPi) was performed [17,18,19]. There are also instances of DBS-Vim [20] and GPi-VIM-DBS treatments [21].

Bilateral GPi-DBS has been employed in cases of myoclonic dystonia resulting from mitochondrial disease—specifically complex I deficiency—where a significant improvement was observed immediately following the initiation of stimulation [22]. The research also indicates that GPi-DBS may serve as an effective intervention when the specific mutation responsible for myoclonic dystonia remains unidentified, provided SGCE gene abnormalities have been excluded [23]. A meta-analysis involving 71 patients with various forms of myoclonic dystonia demonstrated improvement with DBS, as evidenced by an average increase of 79.5 ± 18.2 in both the BFMDRS (Burke–Fahn-Marsden Dystonia Rating Scale) and UMRS (Unified Myoclonus Rating Scale) [24]. Considering all patients with any type of dystonia, the most substantial benefits from DBS treatment are observed in young individuals with primary dystonia, a brief disease history, and mild symptom severity [25]. Furthermore, the presence of the DYT-1 mutation is associated with a highly favorable response to DBS treatment [25]. The prevalence of primary dystonia is estimated at approximately 16.4 per 100,000 individuals [26]. Although this figure may be underestimated, the condition is regarded as rare. According to Orphanet data, the prevalence of myoclonic dystonia is even lower, at approximately 1 in 500,000 individuals. Due to the limited number of patients and unequal access to specialized centers, data on the outcomes of surgical treatment in this patient group are sparse and inconclusive, relying primarily on case reports from individual centers and review articles summarizing their findings. Consequently, each account of a patient with myoclonic dystonia treated with GPi-DBS is invaluable for evaluating the therapeutic potential in this cohort [27]. This is particularly pertinent when the precise genetic mutation causing the symptoms is known, a discovery made only a few years ago. The case described herein represents the first successful attempt at DBS Gpi in a patient with myoclonic dystonia with a confirmed KCNN2 gene mutation, and it was successful. This offers a promising outlook for future patients; however, further studies are necessary for a comprehensive assessment in this context.

## 4. Conclusions

The case of our patient suggests that bilateral DBS-GPi surgery in individuals with DYT 34, characterized by a KCNN2 gene mutation, may significantly reduce dystonic and myoclonic movements in the head, neck, and limbs over the long term. Therefore, genetic testing in patients with dystonia is crucial to determine the appropriate treatment course in cases of genetically determined dystonia, including those with KCNN2 gene mutation. This recommendation aligns with the guidelines of the European Federation of Neurological Societies [4].

## Data Availability

The original contributions presented in this study are included in the article. Further inquiries can be directed to the corresponding author.

## Supplementary Materials

The following supporting information can be downloaded at: https://www.mdpi.com/article/10.3390/ijms26167736/s1.
