# Supplementary material for: Long-Term Efficacy of Bilateral Globus Pallidus Internus Deep Brain Stimulation in Myoclonus-Dystonia Associated with KCNN2 Gene Mutation: A Case Study
Source: Int J Mol Sci. 2025 Aug 10;26(16):7736. doi: 10.3390/ijms26167736 (PMC12386642; doi:10.3390/ijms26167736)
Supplement: Supplementary file 1 [file ijms-26-07736-s001.zip › ijms-3770980-supplementary.pdf]

Suppl 1. The list of genes included in dystonia panel.

ADAR, ADCY5, ANO3, ATM, ATP1A3, ATP7B, BCAP31, CACNA1A, COASY, COL6A3, COX20, DNAJC12, FA2H, FBXO7, FTL, GCDH, GCH1, GNAL, HPCA, KCD17, KCNA1, KCNMA1, KIF1C, KMT2B, MECR, PANK2, PINK1, PLA2G6, PNKD (MR1 – ex1), PRKN (PARK2), PRKRA, PRRT2, SCN8A, SGCE, SLC19A3, SLC2A1, SLC30A10, SLC39A14, SLC6A3, SPR, TAF1, TH, THAP1, TOR1A, TUBB4A, VAC14, VPS13A

Suppl. 2. Summary of KCNN2 patients.

Author	When	Patients	Age	Symptoms	Treatment	Comments
V. Raghuram et al. [13]	2017	1	43	Tonic–clonic seizures, ataxia of gait, stiff legs, dysarthria, confined to a bed at age 42	Levodopa/ carbidopa, baclofen	Also *ZNF135* mutation
B. Balint et al. [10]	2020	5	17/31/35/ 39/63	Head tremor, torticollis, postural jerky hand tremor, writing difficulties, end-gaze nystagmus	Clonazepam 0.25 mg in 35-yo	Family
F. Mochel et al. [11]	2020	11	2/5/7/9/ 15/16/17/ 30/60	Motor and language developmental delay, intellectual disability, behavioural disturbances, cerebellar ataxia and/or extrapyramidal symptoms, myoclonus-dystonia in 17-yo	Not mentioned	Patients unrelated, mostly de novo mutation
B. Lavenstein et al. [28]	2022	1	10	Developmental delay, postural and kinetic tremors, myoclonic jerks in the eyelids, trunk, and bilateral upper extremities, gait disturbance	Not mentioned	No KCNN2 mutation in parents
C. Fearon et al. [29]	2022	1	19	Developmental delay, right torticollis with left laterocollis, upper limb tremor, myoclonic jerks of upper limbs, lower limbs and trunk.	Not mentioned	
M. d'Apolito et al. [12]	2023	4	15/40/42/ 70	Upper extremity tremors, anxiety	Not mentioned	Family
